# Supplementary material for: The Role of Dietary Patterns and Dietary Quality on Body Composition of Adolescents in Chinese College
Source: Nutrients. 2022 Oct 28;14(21):4544. doi: 10.3390/nu14214544 (PMC9654524; doi:10.3390/nu14214544)
Supplement: Supplementary file 1 [file nutrients-14-04544-s001.zip › nutrients-1974656-supplementary.pdf]

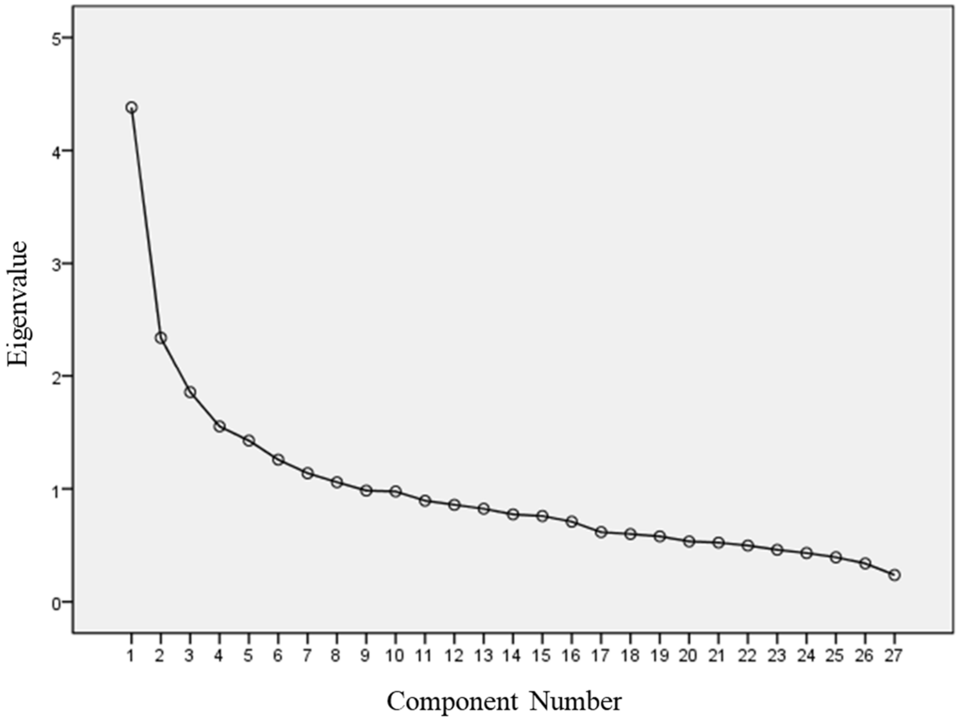

Figure S1. Scree plot of eigenvalues resulting from principal component analysis.

Table S1. Mean or medium number of both with positive and negative fractions servings consumed per day with scores in the top quartile of different dietary patterns.

	RNIs/AIs	Pattern 1	Pattern 2	Pattern 3	Pattern 4	p
Cereal, g/d	200~300 g/d					
HBS		332.0 (246.3,422.4)	361.7 (255.1,477.3)	372.4 (250.1,504.9)	346.0 (228.4,460.5)	0.408
LBS		180.3 (133.3,257.1)	211.0±92.9	195.3 (148.9,258.1)	179.5 (125.3,241.3)	0.536
Red meat and products and poultry	300~500 g/w					
HBS		160.0 (110.5,242.9)[c]	151.8 (88.6,224.3)[c]	210.5 (136.9,300.0)[ab]	160.5 (121.0,250.0)	0.000**
LBS		43.6±21.2	56.9±28.1	58.1±27	53.9±30.4	0.624
Red meat-derived protein, g/d		13.8(6.9,17.4)[c]	8.7(6.9,17.4)[cd]	17.4(13.8,34.8)[ab]	17.4(8.7,18.2)[b]	0.000**
Poultry-derived protein, g/d		5.8(4.1,11.5)	5.8(2.8,9.7)[c]	9.1(4.6,11.5)[b]	5.8(2.9,11.5)	0.022*
Processed meat-derived protein, g/d		1.9(0.6,4.5)[bd]	0.6(0,1.9)[ac]	1.9(0.6,4.5)[bd]	1.2(0.2,3.7)[ac]	0.000**
Egg, g/d	300~350 g/w					
HBS		50(50,50)[d]	50(50,50)	50(50,100)	50(50,100)[a]	0.000**
LBS		29(10.5,50)[d]	39.5(25,50)	25(10.5,50)[d]	50(25,50)[ac]	0.004**

HBS, high bound score; LBS, low bound score; RNI: recommended nutrient intake; AI: adequate intake. d, day; w, week. *p<0.05, **p<0.01. [a]$p < 0.05$, statistically different with pattern 1; [b]$p < 0.05$, statistically different with pattern 2; [c]$p < 0.05$, statistically different with pattern 3; [d]$p < 0.05$, statistically different with pattern 4.
